# Stigmatization in the context of the COVID-19 pandemic: a survey experiment using attribution theory and the familiarity hypothesis

**DOI:** 10.1186/s12889-023-15234-5

**Published:** 2023-03-18

**Authors:** Sebastian Sattler, Dina Maskileyson, Eric Racine, Eldad Davidov, Alice Escande

**Affiliations:** 1 Faculty of Sociology, Bielefeld University, Bielefeld, Germany; 2 Institute of Sociology and Social Psychology, University of Cologne, Cologne, Germany; 3 Pragmatic Health Ethics Research Unit, Institut de Recherches Cliniques de Montréal, Quebec, QC Canada; 4 Department of Medicine, Université de Montréal, Quebec, QC Canada; 5 Department of Neurology and Neurosurgery, McGill University, Quebec, Canada; 6 University of Zurich and University Research Priority Program “Social Networks”, Zurich, Switzerland; 7 Behavioural Insights Team, Paris, France

**Keywords:** Stigmatization, Infectious disease, Pandemic, COVID-19, Flu, Knowledge, Attribution theory, Familiarity hypothesis, Survey experiment

## Abstract

**Background:**

The COVID-19 pandemic has created a global health crisis, leading to stigmatization and discriminatory behaviors against people who have contracted or are suspected of having contracted the virus. Yet the causes of stigmatization in the context of COVID-19 remain only partially understood. Using attribution theory, we examine to what extent attributes of a fictitious person affect the formation of stigmatizing attitudes towards this person, and whether suspected COVID-19 infection (vs. flu) intensifies such attitudes. We also use the familiarity hypothesis to explore whether familiarity with COVID-19 reduces stigma and whether it moderates the effect of a COVID-19 infection on stigmatization.

**Methods:**

We conducted a multifactorial vignette survey experiment (2^8^-design, i.e., *N*_*Vignettes*_ = 256) in Germany (*N*_*Respondents*_ = 4,059) in which we experimentally varied signals and signaling events (i.e., information that may trigger stigma) concerning a fictitious person in the context of COVID-19. We assessed respondents’ cognitive (e.g., blameworthiness) and affective (e.g., anger) responses as well as their discriminatory inclinations (e.g., avoidance) towards the character. Furthermore, we measured different indicators of respondents’ familiarity with COVID-19.

**Results:**

Results revealed higher levels of stigma towards people who were diagnosed with COVID-19 versus a regular flu. In addition, stigma was higher towards those who were considered responsible for their infection due to irresponsible behavior. Knowing someone who died from a COVID infection increased stigma. While higher self-reported knowledge about COVID-19 was associated with more stigma, higher factual knowledge was associated with less.

**Conclusion:**

Attribution theory and to a lesser extent the familiarity hypothesis can help better understand stigma in the context of COVID-19. This study provides insights about who is at risk of stigmatization and stigmatizing others in this context. It thereby allows identifying the groups that require more support in accessing healthcare services and suggests that basic, factually oriented public health interventions would be promising for reducing stigma.

**Supplementary Information:**

The online version contains supplementary material available at 10.1186/s12889-023-15234-5.

## Background

Infectious contagious diseases are by their very nature diseases that are associated with human social behaviors. The fact that human behaviors influence the spread of these diseases implies that they can therefore become the target of public health attention and interventions. A common accompaniment is that infectious contagious diseases can become highly stigmatize﻿d [1], that is, people suffering from them or spreading them through their behaviors can be marginalized or criminalized because of the harm induced to others.

The role of stigma in public health is controversial. Stigmatization can generate an apparently justified negative labeling to behaviors like tobacco smoking, drug use, and risky behaviors [2], and has even been shown to be effective in reducing certain harmful behaviors, such as tobacco smoking [3]. However, stigma can harm and be counterproductive because it can undermine support seeking and the adequate provision of health services to those affected, negatively affecting their health [4, 5]. It can also lead to reactance, which can reduce compliance with pandemic containment rules, as well as refusal to vaccinate.

The current COVID-19 outbreak has provoked stigma and discriminatory behaviors against people who are perceived to carry the virus, especially for people of certain ethnic backgrounds (e.g., Asian) as evidenced by early observations [6]. Many media outlets around the globe have also reported cases of discrimination and racism (e.g., [7, 8]). Previous studies suggested that anxiety and concern of being discriminated against can reduce the use of health services of symptomatic COVID-19 patients, leading to a delay in diagnosis and the rapid spread of COVID-19 in communities [9]. Thus, stigma associated with infectious diseases is also an important social determinant of health behavior [10, 11], and understanding its causes can contribute to improving population health.

Through the lens of attribution theory and familiarity hypothesis [12, 13], we use an experimental vignette design to explore: (1) whether a character diagnosed with COVID-19 is stigmatized more (versus the flu); (2) the role of further disease-related and individual attributes of this person (e.g., reckless behavior); (3) whether the effects of these attributes are stronger for a diagnosis of COVID-19 compared to the flu; (4) the impact of familiarity with COVID-19; and (5) whether familiarity reduces possible effects of stigmatizing attitudes towards a COVID-19 diagnosis (versus the flu).

The contribution of this study is twofold: From a research standpoint, it aims at improving our understanding of the mechanisms that underlie stigmatization in the context of infectious contagious diseases like COVID-19. From a practical standpoint, this study sheds light on who is most susceptible to being stigmatized in the context of COVID-19 (at the time of data collection), and thereby allows identifying the groups that need more support in accessing healthcare services in similar situations in the future. Furthermore, the findings facilitate the assessment of who is more likely to stigmatize and therefore help identify ways to reduce stigmatizing behaviors in those who would be more likely to do so.

### Understanding stigmatization in the context of COVID-19

#### Assumptions based on attribution theory

Attribution theory has long been applied to stigmatization in the context of mental illnesses and infectious diseases [12, 14–17]. This theory postulates that people attribute different levels of controllability, responsibility, and dangerousness to others’ actions to help them understand and explain the causes of the conditions, behaviors, and consequences of these actions [12, 18]. Accordingly, people search for or are receptive to signals and signaling events that shape their cognitive beliefs (e.g., blameworthiness and dangerousness), affective reactions (e.g., anger and fear), and discriminatory inclinations (e.g., coercion, avoidance, segregation, or withholding help). In the general context of infectious diseases, and COVID-19 in particular [19, 20], such signals and signaling events include information about whether the onset of the disease is rooted in the person’s (irresponsible) behavior, as opposed to being caused coincidentally or by others. According to the theory, attributions of higher control over the onset of a disease may in turn increase stigma. In addition, the reckless behavior of a potentially infected individual (e.g., refusing to go into quarantine) implies running the risk of infecting others and would be perceived negatively.

This study examines eight factors that may form partially interwoven signals and signaling events affecting stigma towards a person infected with COVID-19. These factors have received various levels of empirical scrutiny and support [12, 20–23]. However, their effect on stigmatization in the context of COVID-19 has hardly been studied. In this study, we therefore examine whether and to what extent the following disease-related and individual factors are associated with higher or lower stigmatization in the context of a COVID-19 infection.

Diagnosis. Since COVID-19 is an infectious disease that can be controlled in-part by one’s behavior (i.e., via adherence to preventive behavioral measures), and a disease that is perceived as considerably more dangerous than a seasonal flu [24, 25], we expect more negative cognitions, affects, and discriminatory inclinations towards those infected with it.

Precipitating event. Stigmatizing attitudes are likely to be stronger towards people who may be considered responsible for getting infected [20]. That is, if the infection is due to one’s own behavior instead of having contracted the disease through the behavior of others, individuals should be stigmatized more.

Reckless behavior. The violation of policy regulations that serve to mitigate the spread of the virus (e.g., quarantine restrictions, social distancing) might be perceived as reckless and dangerous behavior [26]. Such behavior should elicit more stigma compared to adherence to policy regulations.

At-risk group membership. Individuals belonging to a risk group may elicit benevolence stigma [27], which is the perception of needing to be taken care of, like a child. This perception could lead to less negative cognitions and affects towards this group, but potentially higher stigmatizing behaviors, such as avoidance. At the same time, at-risk group members might be generally sicker and may thus be perceived as a burden to the healthcare system and society [28], and consequently suffer from greater stigma.

Risk area. Potentially, persons from a high COVID-19 incidence area might be considered responsible for the high incidence rate, and might be seen as more at-risk for the contamination and spread of it compared to people from areas with a lower incidence rate [29]. This can increase stigmatization, also as a self-protection mechanism. However, individuals from such areas might also be seen as having less control of contraction and therefore less responsible for their infection.

Gender. In line with previous studies, we expect stigma to be lower towards females because they are the object of benevolence stigma [23, 26, 30, 31]. Males might be subject to higher stigma due to the gendered ideas of them being invulnerable, strong, independent, etc., and due to the fact that they are more likely to not comply with behavioral recommendations and thus act more irresponsibly. Indeed, previous studies demonstrated that men were more likely to hold irresponsible attitudes towards the COVID-19 pandemic, which could indicate a lower consciousness about the potential danger of the virus [32, 33]. Conversely, males may receive less stigma, because they often suffer from a more severe course of the illness (than females) [34, 35], which could also elicit benevolence stigma towards them.

Age. Older people may also face benevolence stigma or “compassionate ageism,” due to their higher vulnerability and belonging to a risk group compared to young people [27], which may result in less stigma. Conversely, they may suffer from greater stigma since older people could be perceived as sicker, cognitively impaired, and a higher economic burden on society because of their growing consumption of health and welfare services [36, 37]. Other studies have shown that older people have been rated as less responsible compared to younger people [38].

Origin. Prejudice towards foreigners may translate not only into more negative attitudes towards seasonal foreign workers in general (compared to German citizens who may be considered by respondents as more familiar), but also to higher stigma towards them in the context of COVID-19 [19, 39]. One reason could be that these prejudices may include blaming these people for the spread of the disease.

Interaction effects between COVID-19 diagnosis and disease-related and individual attributes. In contrast to the seasonal flu, COVID-19 is a threatening virus with more severe consequences to infected adults [24, 25]. We want to explore whether the expected effect of the diagnoses (COVID-19 versus seasonal flu) varies across disease-related and individual attributes on stigmatizing attitudes. We expect the effects of these attributes to be stronger if the diagnosis of an infected individual is COVID-19 compared to a diagnosis of a seasonal flu. For example, a COVID-19 infection due to a precipitating event for which an individual could be seen as responsible should increase stigma as compared to an onset event of the disease for which the individual is not seen as responsible.

#### Assumptions based on familiarity hypothesis

According to the familiarity hypothesis [12, 40], respondents’ familiarity with COVID-19 can influence stigmatizing attitudes. Tolerance and understanding towards people with an infectious disease might be a function of the self-reported and factual knowledge about the specific disease^1^ or vicarious experience with it (e.g., knowing someone who died from the illness), both being referred to as “familiarity” with COVID-19. Indeed, knowledge and information may reduce threat from the unknown. Studies have found a stigma-inhibiting effect of familiarity in different contexts of stigmatization such as towards people with mental illnesses [23, 40]. For example, a study by Angermeyer, Matschinger, and Corrigan [41] demonstrated that respondents who were familiar with mental illnesses were less likely to believe that people with schizophrenia or major depression were dangerous. In the context of infectious disease, Cotler et al. [42] have identified that higher familiarity and knowledge regarding hepatitis B is correlated with lower stigma scores. A recent field experiment [39] in India found that an information brief decreased the stigmatization of COVID-19 patients and certain groups, such as religious minorities or frontline workers. It also reduced the belief that infection cases are more prevalent among certain marginalized social and economic groups. Indeed, knowledge about COVID-19 should help to better understand that nobody is immune, and thus people with more knowledge and experience may stigmatize less compared to people without. Thus, we expect that familiarity with COVID-19 (i.e., self-reported and factual knowledge about the disease, as well as experience with COVID-19 by knowing someone who died due to COVID-19) lowers stigmatizing.

#### The interplay between COVID-diagnosis and COVID-19 familiarity

While attribution theory focuses on how stigmatizing cognitions, affects, and behavior are affected by signals and signaling events in a given situation, the familiarity hypothesis puts emphasis on a person’s familiarity with the event or situation as a potential moderator of the effects of these signals and signaling events on stigmatization. Based on the person–situation debate [43–45], which tackles the question whether social situations, personal reality, or both matter for a person’s reaction in a certain context, studies addressing not only additive but also interactive effects could also improve our understanding of stigmatization in the context of COVID-19. We argue that a more complete analysis of who is stigmatizing in response to signals and signaling events offers practical insights about how situations could be changed as well as who should be approached. Therefore, we also aim to examine whether stigmatization towards people with a COVID-19 diagnosis varies with respondents’ familiarity. Indeed, it could be the case that the inhibiting effect of information and knowledge may not only decrease stigma per se, but also that people with higher familiarity may react differently to the information that a person is infected with COVID-19 (versus the flu) [46]. For example, if people have a vicarious experience with COVID-19 by knowing someone who died, they could better understand that nobody is immune and may react differently to this information. Thus, we want to explore whether the stigma-increasing effect of a COVID-19 diagnosis on stigma is weaker at higher levels of familiarity with COVID-19.

## Methods

### Data

To examine stigma in the context of COVID-19, we recruited 4,856 adult participants in Germany via an online panel from the survey provider Respondi. In an elaborate scoring and control process, this online panel is subjected to continuous quality control. We used a quota sample that was representative for sex, age, and federal state of the adult population (18–74 years old) in Germany. Data were collected between December 16 and December 29, 2020. During this time the country was under a lockdown, hence during which non-essential stores and services as well as schools were closed. In accordance with German data protection regulations, personal data and survey data were stored separately. Researchers have no access to personal data and are thus, not able to identify respondents. Panel members were invited via an email in which the topic was not mentioned, thereby reducing selective survey uptake due to topic salience. Of the 4,856 participating respondents, 4,716 participants (97.1%) provided informed consent. After omitting individuals who either refused to participate or skipped questions, the final sample size comprised of 4,059 participants (females 49.5%; average age: 45.68 years) who provided answers to all questions in the survey. Respondents completing the survey received a small incentive (€0.40) consistent with the payment modalities of Respondi.

### Factorial survey design

We pursued a full factorial survey design with vignettes (i.e., short descriptions of situations) [47–49]. We varied experimentally and simultaneously, characteristics of a fictitious person who possibly contracted a COVID-19 virus, the circumstances of contraction, and how the person behaved in that context. The use of multi-factorial designs allows for a causal interpretation of effects of the variation of multiple experimental treatment variables on the outcome variables. The advantage of the survey experiments using vignettes is that they provide higher internal validity (due to the orthogonal design and the controlled setting) than classical surveys and also higher external validity (due to a large, diversified sample) than many lab experiments. Vignettes are useful to study stigmatization because they can be a defendable substitute for ethically and practically challenging real world manipulations [50, 51] and reduce social desirability bias [52, 53]. Studies have also shown that the effects estimated with vignette designs remarkably matched the effects found in other research designs [54, 55].

In the current study, we randomly and simultaneously varied eight factors with two levels each (see Table 1). This resulted in a 2^8^ design (*N*_*Vignettes*_ = 256): gender; age; origin; living in a COVID-19 risk area; precipitating event of contracting an infection; belonging to a COVID-19 risk group (defined as having a previous illness); violation of quarantine instructions; and diagnosis of disease. Each respondent was randomly assigned to one vignette, leading to a between-subjects design that avoided potential learning, contrast, and fatigue effects [48, 56]. Due to the factorial design, all factors are uncorrelated, that is, all correlations between these eight factors were very weak (with a maximum correlation of *r* <|0.051|). Due to the random allocation of vignettes to respondents, vignette factors and respondent characteristics are also uncorrelated (*r* <|0.041|).

**Table 1:** Vignette dimensions and levels. Experimental variation of eight dimensions (*N*_*Vignettes*_ = 256)

Dimension	Levels	Examples of two contrasting vignettes
Gender	▪Male ▪Female	*Example for males:* • **Alexander** is **21** years old. You do not know **Alexander**. • **He** works as a **harvester** on a farm in Germany. • **He** currently lives in a county where Corona **hardly occurs**. • Recently, **Alexander** spontaneously visited a friend with whom **he** wanted to have a coffee. • Because **Alexander** developed a slight cough a few days later, **he** went to **his** family doctor. The doctor ordered a Corona test. • **However, Alexander does not have any relevant previous illnesses**. • While **Alexander** was waiting for the test results, **he went** into domestic quarantine as prescribed. • Today, the results came back. They confirmed that **he** does **not have Corona, but only the flu**. *Example for females:* • **Alexandra** is **61** years old. You do not know **Alexandra**. • **She** works as a **foreign seasonal harvester** on a farm in Germany. • **She** currently lives in a county where Corona is **very widespread**. • Recently, **Alexandra** spontaneously visited a friend with whom **she** wanted to have a coffee. **She knows that the friend is currently waiting for his test results and has a few Corona symptoms**. • Because **Alexandra** developed a slight cough a few days later, **she** went to **her** family doctor. The doctor ordered a Corona test. • **In fact, Alexandra has a relevant pre-existing condition (cardiovascular problems)**. • While **Alexandra** was waiting for the test results, **she, however, did not go** into domestic quarantine as prescribed. • Today the results came back. They confirmed that **she** does **have Corona**.
Age	*▪*Young *▪*Old	
Origin	*▪*Local ▪Foreigner	
Risk area	*▪*No ▪Yes	
Precipitating event of contraction	*▪*Blank ▪Visited friend	
Belonging to risk group	*▪*No ▪Yes	
Quarantine instruction	*▪*Followed ▪Violated	
Diagnosis	*▪*Flu ▪COVID-19	

*Note:* The text in bold indicates the varied vignette dimensions (not bolded in the survey)

### Measured variables

**Fig. 1:**
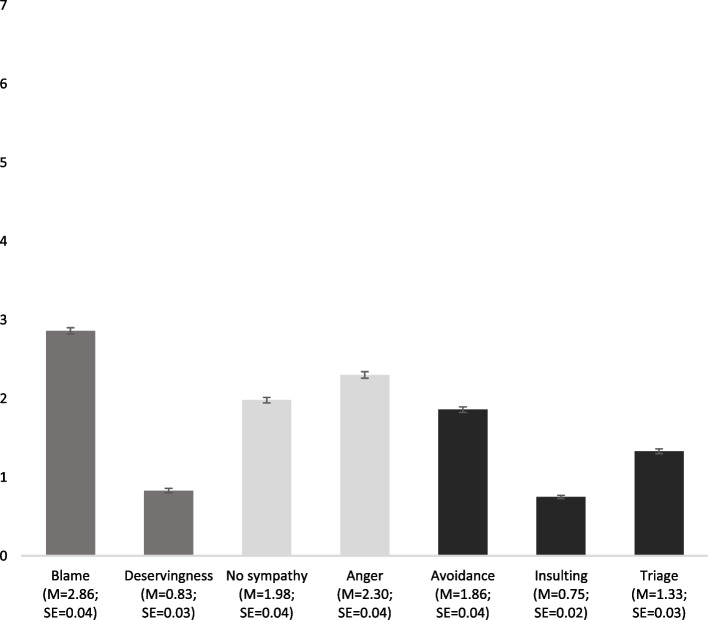
Means (M) and standard errors (SE, error bars) of  negative cognitions,  negative affects, and  negative behavioral inclinations (*N*_*Participants*_ = 4,059). *Notes: Blame:* I am of the opinion that *Alexander himself/Alexandra herself*^‡^ is to blame for having *flu/Corona*^**†**^. *Deservingness: Alexander/Alexandra*^‡^ deserves to have *flu/Corona*^**†**^. *No sympathy:* I would have no sympathy for Alexander/Alexandra^‡^. *Anger:* I would be angry with *Alexander/Alexandra*^‡^. *Avoidance:* Even after *Alexander's/Alexandra's*^‡^ recovery, I would avoid meeting *him/her*^‡^. *Insulting:* I would insult *Alexander/Alexandra*^‡^ if I saw *him/her*^‡^. *Triage:* If hospital treatment capacity were limited, *Alexander/Alexandra*^‡^ should be given a lower priority for future illnesses. ^‡^The displayed gender aligned to the gender in the vignette. ^**†**^The displayed illness aligned to the illness in the vignette. Responses were assessed on a scale from “*does not apply at all*” (0) to “*fully applies*” (7)

Stigmatizing attitudes: Seven items measured stigma [57–59] after people read the vignette (see Fig. 1 for item texts): Two items assessed negative cognitions (i.e., beliefs), namely, the perception of if the vignette character should be blamed for contraction of either the flu or COVID-19, and the perception of if the vignette character deserves to be infected. Negative affect was assessed with two items, having no sympathy for and feeling angry at the person described in the vignette. Three items measured discriminatory inclinations: avoidance (the intention to stay away from the vignette character after recovery), the willingness to insult the vignette character, and finally, triage (if the vignette character should be given a lower priority in the future if hospital capacity was limited). The eight-point response scale for these seven items ranged from “does not apply at all” (0) to “fully applies” (7).

Familiarity: We measured COVID-19 familiarity with three measures. Vicarious experience with a lethal outcome of a COVID-19 infection was measured by asking the question: “Do you know someone personally who died either directly or indirectly from Corona?”. Response options were “no” (0) and “yes” (1). Self-reported COVID-19 knowledge about the symptoms and consequences of COVID-19 was measured with the item “My knowledge of the symptoms and effects of Corona is…” “very low” (0) to “very high” (10) [23]. Factual COVID-19 knowledge was assessed in a brief COVID-19 knowledge test with eight yes/no questions, such as, “Is dry cough a symptom of corona?” or “Is corona caused by a bacterium?” (see Supplementary Information, Table S1 for all items). The variable was constructed as a count index for which only correct answers counted (see Table 2). For the sake of interpretation of the interaction analysis, we normalized the knowledge measures to a (0, 1) interval so that “0” indicated a respondent with no knowledge and “1” indicated a respondent with high knowledge, having answered all questions correctly (any other level of knowledge was between 0 and 1).

**Table 2:** Correlations and descriptive statistics of respondents’ characteristics (*N*_*Participants*_ = 4,059)

Respondent characteristic	Pearson’s correlation coefficients						Descriptives			
	1	2	3	4	5	6	***M***	***SD***	***Min***	***Max***
1: Female (Ref. Male)	1.00						1.49	0.50	0	1
2: Age	0.03	1.00					45.68	15.49	18	74
3: Self-reported COVID-19 knowledge^‡^	0.07^***^	-0.09^***^	1.00				7.19	1.86	0	10
4: COVID-19 knowledge test^‡^	0.08^***^	0.05^***^	0.21^***^	1.00			7.16	1.21	0	8
5: Vicarious experience (Ref. None)	0.01	-0.07^***^	0.05^**^	-0.05^**^	1.00		0.09	0.28	0	1
6: Anonymity perceptions	0.07^***^	0.14^***^	0.15^***^	0.18^***^	0.03	1.00	6.19	1.39	0	7

***Notes:*** *p < .05, **p < .01, *** p < .001. *M*  Mean, *SD*  Standard deviation, *Min*  Minimum, *Max*  Maximum. ^‡^Before transformation

### Pretesting

We pretested the vignettes and all other materials by conducting think-aloud cognitive pretests with probing questions (*N* = 10) to evaluate and improve the instruments [60]. We adjusted the vignette following the responses of the pretest subjects. For example, items with no variance (e.g., perceiving hate towards the vignette character) were deleted or slightly changed.

### Statistical analysis

Negative binomial regression models [61] were computed to estimate the effects of the independent variables (i.e., the eight vignette dimensions, three indicators of familiarity, and respondents’ gender and age) on prejudice. This class of models is appropriate due to the overdispersion (i.e., variance is greater than the mean) of the outcome variables and should therefore produce more efficient, consistent, and less biased estimates than ordinary least squares approaches [62]. We observed overdispersion in our data, also indicated by statistically significant likelihood-ratio chi-square tests estimating whether the dispersion parameter alpha is equal to zero for our seven outcome variables.

In the first set of analyses, we examined the main effects of all variables under investigation. In a second set of analyses, we tested our assumptions regarding interaction effects between COVID-19 diagnosis and disease-related and individual attributes as well as with COVID-19 familiarity by adding interaction terms between COVID-19 diagnosis and all other variables under investigation. In all analyses we also show the effects of the respondents’ gender and age. Moreover, we controlled for perceived anonymity of the survey [63], because a lack of anonymity is an important risk factor for biased answers due to social desirability when asking about sensitive topics [64]. Anonymity perceptions were assessed with one item asking respondents to assess, on a response scale ranging from “does not apply at all” [0] to “fully applies” [7], their perceived trust in the confidentiality of their answers.

We reported incidence rate ratios (IRR). Thereby, an IRR greater than 1 indicates a positive effect of the independent variable on the outcome variable, while an IRR smaller than 1 reflects a negative effect, and an IRR equal to 1 indicates no effect. We present IRRs along with *p*-values for readers interested in one of the seven specific outcomes, but due to multiple hypothesis testing with seven outcomes, we rely on the more rigorous sharpened false discovery rate (FDR, i.e., the expected proportion of rejections of any type-1 errors) *q*-values to indicate statistical significance in the Results section [65]. This approach “formalizes the trade-off between correct and false rejections and reduces the penalty to testing additional hypotheses.” [65]. However, the majority of results with unadjusted *p*-values and sharpened FDR-adjusted *q*-values appear to be essentially similar (see Tables 3 and S2).

**Table 3:** Multivariate negative binomial regression models^†^ on stigma in the context of COVID-19 (*N*_*Participants*_ = 4,059)

**Model**		**1**	**2**	**3**	**4**	**5**	**6**	**7**
**Stigma type**		**Negative cognition**		**Negative affects**		**Discriminatory inclinations**		
**Stigma facet**		**Blame**	**Deservingness**	**No sympathy**	**Anger**	**Avoidance**	**Insulting**	**Triage**
**Vignette characteristics**								
Diagnosis: COVID-19 (Ref. Flu)	*IRR*	1.923	1.203	1.248	1.469	1.258	1.182	1.233
	*p*	.000***	.007**	.000***	.000***	.000***	.020*	.000***
	*q*	.001**	.014*	.001**	.001**	.001**	.032*	.001**
Precipitating event: Visited friend (Ref. Blank)	*IRR*	1.787	1.828	1.690	1.724	1.511	1.540	1.570
	*p*	.000***	.000***	.000***	.000***	.000***	.000***	.000***
	*q*	.001**	.001**	.001**	.001**	.001**	.001**	.001**
Quarantine instruction: Violated (Ref. Followed)	*IRR*	1.256	1.638	1.768	2.069	1.462	1.790	1.573
	*p*	.000***	.000***	.000***	.000***	.000***	.000***	.000***
	*q*	.001**	.001**	.001**	.001**	.001**	.001**	.001**
Belonging to risk group: Yes (Ref. No)	*IRR*	1.025	1.007	1.024	1.025	1.108	1.043	.994
	*p*	.413	.923	.566	.513	.022*	.556	.907
	*q*	.367	.591	.445	.421	.033*	.442	.588
Risk area: Yes (Ref. No)	*IRR*	1.025	1.081	1.076	1.060	1.072	1.056	1.004
	*p*	.416	.259	.069	.128	.121	.447	.941
	*q*	.368	.259	.086	.144	.136	.390	.594
Gender: Female (Ref. Male)	*IRR*	.949	.851	.915	.927	.878	.811	.892
	*p*	.084	.020*	.029*	.049*	.004**	.004**	.041*
	*q*	.102	.031*	.041*	.063	.008**	.008**	.056
Age: Old (Ref. Young)	*IRR*	1.003	.977	1.058	.994	1.063	.945	1.045
	*p*	.913	.735	.164	.883	.171	.428	.426
	*q*	.588	.515	.177	.588	.184	.375	.375
Origin: Foreigner (Ref. Local)	*IRR*	.985	1.037	.946	.946	.969	.901	.963
	*p*	.626	.600	.168	.145	.484	.146	.497
	*q*	.483	.466	.181	.159	.411	.159	.413
**Familiarity indicators**								
Self-reported COVID-19 knowledge	*IRR*	1.359	1.403	1.347	1.738	1.349	1.314	1.430
	*p*	.000***	.085	.009**	.000***	.016*	.177	.027*
	*q*	.001**	.102	.016*	.001**	.026*	.189	.039*
COVID-19 knowledge test	*IRR*	.671	.179	.653	.623	.425	.149	.315
	*p*	.000***	.000***	.001**	.000***	.000***	.000***	.000***
	*q*	.001**	.001**	.004**	.001**	.001**	.001**	.001**
Vicarious experience (Ref. None)	*IRR*	1.049	1.043	1.183	1.185	1.133	1.383	1.248
	*p*	.363	.730	.017*	.010*	.109	.009**	.022*
	*q*	.345	.515	.027*	.019*	.126	.016*	.033*
**Further respondent characteristics**								
Female (Ref. Male)	*IRR*	.883	.723	.876	.970	.919	.745	.742
	*p*	.000***	.000***	.001**	.429	.060	.000***	.000***
	*q*	.001**	.001**	.003**	.375	.075	.001**	.001**
Age	*IRR*	.993	.991	.994	.991	.995	.999	.993
	*p*	.000***	.000***	.000***	.000***	.000***	.741	.000***
	*q*	.001**	.001**	.001**	.001**	.001**	.515	.001**
**Anonymity perceptions**	*IRR*	1.002	.913	.960	1.002	.977	.882	.944
	*p*	.870	.001***	.009**	.877	.175	.000***	.009**
	*q*	.588	.002**	.017*	.588	.188	.001**	.017*
**Constant**	*IRR*	2.321	6.154	2.299	1.475	2.962	6.360	4.928
	*p*	.000***	.000***	.000***	.012*	.000***	.000***	.000***
	*q*	.001**	.001**	.001**	.021*	.001**	.001**	.001**

***Notes:***^†^Incidence rate ratios (sharpened false discovery rate-adjusted q-value in parentheses). * *p & q* < .05; ** < 0.01; *** < 0.001 (two-tailed)

## Results

### Descriptive results

Figure 1 displays the mean levels of the stigma measures across the different vignettes. Respondents were especially inclined to blame the depicted vignette character (20.8% chose one of the two highest response options). However, declaring that this vignette character deserved the illness was not very common (2.8%). Respondents also experienced moderate levels of negative emotions (no sympathy: 9.2%; anger: 15.1%). Even after recovery of the described person, respondents were moderately willing to avoid meeting the vignette character (9.4%), but much fewer would insult them (2.5%). Agreement to restrict access to future healthcare via triage was slightly more common (5.6%). Moreover, self-reported COVID-19 knowledge was relatively high (Table 2), that is, 22.4% chose one of the two highest response options. Half of the respondents (51.4%) provided correct answers to all questions in the knowledge test. However, the two knowledge measures only correlated moderately (*r* = 0.21, *p* < 0.001). Moreover, the vast majority (79.8%) of respondents (very) strongly expected that their responses will remain confidential, while only less than 1.7% expected that confidentiality (absolutely) does not apply, which overall suggests high perceived anonymity.

### Multivariate analysis

This section presents the results of the multivariate negative binomial regression analyses (see Table 3) to discern which factors resulted in higher stigma. Models 1 to 7 show that higher stigma occurred regarding all outcomes (especially the negative cognition blame) if the vignette character was diagnosed with COVID-19 (compared to a seasonal flu). Furthermore, more stigma (again especially blaming the vignette character) was elicited if the vignette character visited a friend while knowing that the friend had COVID-19 symptoms and was waiting for the diagnosis. In the case of violating quarantine rules, the level of all stigma outcomes (especially the negative emotion anger) was elevated. Respondents were more likely to avoid meeting persons belonging to a COVID-19 risk group. Females in the vignettes were subject to lower stigma outcomes (except for blame, anger, and triage, where no statistically significant differences were found) than males. Whether the vignette character was from a high COVID-19 risk area (as compared to not), was a foreigner (as compared to local), and was old (as compared to young) did not impact stigma measures.

Surprisingly, self-reported COVID-19 knowledge was positively associated with higher levels of all forms of stigma (except for deservingness and insulting). However, higher levels of factual COVID-19 knowledge were generally negatively associated with stigma in all its forms. Respondents with vicarious experience with COVID-19 infections (e.g., personally knowing someone who had died from COVID-19, either directly or indirectly), reported more negative emotions and discriminatory inclinations across the vignettes, but they did not display higher levels of blame and deservingness and avoidance.

We also found that female respondents elicited less stigma compared to male respondents in all measures (with the exception of anger and avoidance, where no difference was found). Moreover, older respondents also generally stigmatized less (with the exception of insulting, for which we did not find any differences).

**Fig. 2:**
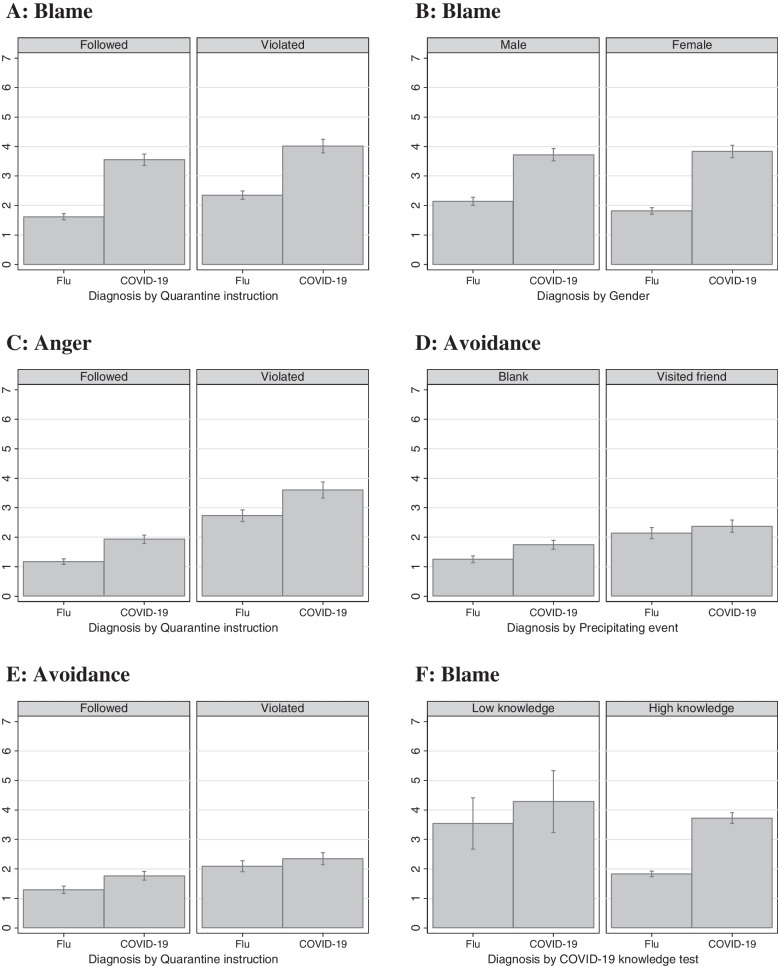
Predicted values (with standard errors) for different stigma indicators depending on COVID-19 diagnosis and further disease-related and individual attributes as well as COVID-19 knowledge test score (*N*_*Participants*_ = 4,059) – based on results in Table S2

We furthermore tested interaction effects between the diagnosis (COVID-19 versus the flu) and each vignette characteristic (see Supplementary Information, Table S2, Models 1 to 7) to explore whether the expected effect of the diagnoses (COVID-19 versus seasonal flu) on stigmatization varies by disease-related and individual attributes. The negative interaction effects between diagnosis and violation of the quarantine instructions suggests that the blame-increasing (Panel A in Fig. 2), anger-increasing (Panel C), and avoidance-increasing (Panel G) effects of a COVID-19 diagnosis are slightly lower if the person violated the instructions as compared to not. Given that blame, anger, and avoidance are relatively high if COVID-19 is diagnosed, a violation of the quarantine instructions can lead to a greater increase in blame, anger, and avoidance in the context of a flu diagnosis as compared to a COVID-19 diagnosis. Panel D shows a slightly stronger effect of a COVID-19 diagnosis on the avoidance of the vignette character in the blank condition, that is, when it was not mentioned that the vignette character visited a friend with COVID-19 symptoms who was waiting for the diagnosis. While both genders diagnosed with COVID-19 received similarly high levels of blame (Panel B), the results suggest that males with flu were blamed more than females. As a result, the difference between a diagnosis of COVID-19 rather than the flu is greater in female as compared to male vignette characters.

Models 1 to 7 in Table S2 shows the results of an exploratory analysis concerning whether the diagnosis (COVID-19 versus the flu) interacts with different indicators of familiarity. They reveal only one statistically significant interaction effect: Respondents who scored higher in the COVID-19 knowledge test hardly blamed a person diagnosed with the flu compared to COVID-19, and respondents with low scores also blamed vignette characters diagnosed with flu and differed less in this blame with regard to a COVID-19 diagnosis (Panel F).

## Discussion

This study aimed at exploring stigmatization in the context of the COVID-19 pandemic in Germany. Thereby, it examined (1) whether a COVID-19 diagnosis (versus the flu) elicits stigmatizing attitudes; (2) the role of disease-related and individual attributes in the formation of stigma; (3) whether the effects of these attributes are stronger for a diagnosis for COVID-19 compared to flu; (4) the impact of familiarity with COVID-19; and (5) whether familiarity can reduce possible stigmatizing effects of a COVID-19 diagnosis (versus the flu).

The descriptive results of our vignette experiment showed that blame was the strongest stigma reaction towards a vignette character with COVID-19, followed by anger. Respondents hardly declared the person deserved the illness and only some were inclined to insult them. Still, few respondents went as far as imposing negative consequences via triage if the vignette character has future health problems, a reaction that could be considered a form of severe punishment.

To understand the links between disease-related and individual attributes and stigmatization, this study utilized attribution theory [12, 18, 31]. Attribution theory explains how people interpret and react to signals and signaling events concerning causes of conditions and the consequences for others. Given that COVID-19 is a more dangerous and infectious disease than the flu [66, 67], and that it is at least partially controllable via careful behavior, we expected higher stigma towards individuals infected by COVID-19 compared to the flu [20, 58]. Indeed, our results corroborate this expectation. Also in line with attribution theory and our expectations, we found that stigma was higher if someone could be held responsible for the onset of their infection due to a precipitating event being under their control [cf., 20]. While respondents generally tended to strongly avoid vignette characters who visited a friend, when no such visit was mentioned in the vignette, a COVID-19 diagnosis elicited a slightly larger effect on avoidance than a diagnosis of the flu. Reckless behavior during a pandemic, in the form of violating quarantine instructions before receiving the diagnosis, increased negative reactions on all outcome variables of stigma [cf., 26]. Due to the already relatively high blame and avoidance of as well as anger towards persons diagnosed with COVID-19, violating quarantine could lead to a higher increase in blame, avoidance, and anger when the flu is diagnosed. Furthermore, respondents reported a higher tendency to avoid individuals belonging to a risk group after their recovery. This could reflect a form of benevolence stigma [27], in which an attempt could be made to avoid putting these group members in a situation that would increase their risk of reinfection due to their higher vulnerability. While one assumption was that persons from a high COVID-19 incidence area are more stigmatized because they could be seen as a high risk for the contamination, spread, and high incidence of the virus, we did not find that they have been subjected to more stigma compared to people not from risk groups.

We found that women were stigmatized less than men (with the exception of blame, anger, and triage, where no statistically significant differences were found). Moreover, we found an interaction effect between gender and diagnosis for blame. While respondents hardly differentiated between males and females infected with COVID-19, females infected with the flu were blamed less compared to males. This finding may provide some empirical support for the existence of benevolence stigma towards these women, in line with other studies [23, 26, 30, 31]. Furthermore, we did not find evidence for stigma of any kind (positive/benevolent or negative) regarding older vignette characters. Similarly, prejudice towards foreigners, such as judging them responsible for the spread of the diseases, was also not found.

This study also sought to discover the relationship between familiarity with COVID-19 and stigma in Germany by relying on the familiarity hypothesis [12, 40]. Consistent with research on stigma and familiarity*—*also in the context of COVID-19 [39]—higher levels of factual COVID-19 knowledge were generally associated with a lower stigma (in five of the seven outcomes). After all, respondents with more factual knowledge about COVID may be more tolerant and understanding towards people with an infectious disease, because they have a better insight into the disease and its sources. This finding supports that education is a powerful and effective tool to develop interventions for combating the stigmatization of individuals infected by COVID.

We also found that respondents with lower knowledge differentiated less in their blaming of persons diagnosed with the flu or COVID-19, as both received relatively high blame, while respondents with more factual knowledge blamed persons diagnosed with the flu less than those with COVID-19. Possibly, people with less knowledge are unaware about the less-severe consequences of the flu compared to COVID-19. Indeed, at the time of our study, there has been rampant false information that COVID-19 is no more harmful than the seasonal flu in addition to similarities between symptoms of these viral infections and government agencies could have also provided better information about differences in case fatality rates [68].

Moreover, our findings revealed that while factual knowledge reduced stigmatization, self-reported COVID-19 knowledge was positively associated with all forms of stigma. This finding is difficult to interpret, especially since knowledge about COVID-19 has become politicized and polarized [69], and shaped by broader social and political identities and discourses [70]. Having the perception of being knowledgeable about COVID-19 may fuel resentment against those who do not know or are careless and ignorant. Given the moderate correlation between both COVID-19 knowledge measures (factual knowledge and self-reported knowledge), it is an interesting question about the meaning of how one evaluates their self-reported knowledge and factual knowledge. Another explanation could be that those who think they know a lot are more willing to make firm judgments and attribute blame and other forms of stigma. An underlying influence could also be personality differences between those who have fixed, strong opinions and those who have more nuanced, humble opinions—i.e., those who may be more fearful of the virus, yet trustful of mainstream science to protect them [71].

On a more speculative note, compared to factual knowledge, self-reported knowledge (about COVID-19) is related to how people evaluate themselves as more knowledgeable or uninformed. It is possible that some people who have less factual knowledge view themselves as being very knowledgeable about COVID-19, an illustration of the well-known and common Dunning–Kruger Effect [72]. The existence of this bias would indicate problematic overconfidence or adherence to dogma about COVID-19, or both [73]. In the latter case, these participants could even reject mainstream factual knowledge but still think they know more or have better information based on alternative information, truths, and facts. This is a common refrain since those who, for example, oppose vaccination think they know more than experts [73]. In the context of the COVID-19 pandemic, the discrepancies between self-reported and factual knowledge could be a manifestation of mistrusting science, mainstream media, and politics because factual knowledge about COVID-19 is associated with trust in science [74] and the use of scientific information [75, 76]. However, one has to be cautious since we only provide correlational evidence between knowledge and the stigmatizing attitudes.

Finally, and in contrast to our expectation, we found that knowing someone who died from COVID-19 led to a higher stigmatization (in four of the seven outcomes). Previous studies show that the availability bias can lead to a higher perceived mortality risk [77]. That is, when people have vicarious experiences and can immediately recall multiple examples of COVID-19 deaths, they might be more likely to believe that such deaths are very common. It could be the case that familiarity with a COVID-19 victim may have reflected retaliation against those who may potentially infect others due to their behavior. This reflects compassion towards the victim who suffered the worst outcome. It reinforces the belief that irresponsible people are causing harm to innocent ones. Higher stigma among respondents familiar with a lethal outcome of COVID-19 might be also due to an elevated fear (for themselves and others) that infected people can transmit the disease to them and thereby put them in danger.

### Strengths, limitations, and outlook for future research

One strength of this study is its basis on a large sample representative for sex, age (18–74), and federal states in Germany, and because prior research on COVID-19 often used smaller, non-representative samples. Another strength is that the use of an experimental vignette design facilitates a causal interpretation of the effects of multiple experimental treatment variables on the outcome variables, it allows factors to be manipulated—which might be challenging in other settings—and it is known to reduce social desirability bias [52, 53]. These features are useful in the study context, for example, because due to the nature of the study, it may be considered socially undesirable to blame, reject, and discriminate against people infected by COVID-19. Therefore, using means to reduce social desirability bias, such as an vignette study design or including perceived anonymity of the survey as a control variable [63], could be seen beneficial. However, the number of “does not apply” responses ranged from 1.7% (insulting) to 4.0% (triage), indicating that such a small fraction of respondents may have used this response category due to social desirability in order to avoid making an assessment, and also perhaps because of the difficulty to judge in this novel pandemic context.

Our study has been conducted during the second wave of the pandemic in Germany characterized by high morbidity and mortality rates as well as a lockdown [78]. Thus, the fear of contracting or spreading COVID-19 and, therefore, negative attitudes towards people infected with COVID-19 might have been more pronounced as compared to the waves of the less severe variant strains. However, at this time the first vaccine “Comirnaty” against COVID-19 was just released in Germany, and a vaccination campaign for defined risk groups had been implemented around the end of the field time of the survey, which could have created some hope, although vaccines only became available to the general population by late summer 2021 [79]. Thus, the time of the survey and the dynamic nature of the pandemic need to be considered when interpreting our results. Future studies may investigate whether and to what extent the stigmatization towards infected individuals is associated with, for example, the current severity of the pandemic and its countermeasures (such as vaccination campaigns).

While this study focused on Germany, it would be vital to conduct further research on the topic in other countries, since the cultural context may shape stigmatization. Such research would be important, because existing evidence demonstrates that aspects of cultures can exacerbate (or ameliorate) the tendency for individuals to stigmatize others [80]. Specifically, group-oriented cultures (e.g., East-Asian countries), in which individual preferences are less important than the norms and needs of groups to which they belong, are characterized by a greater level of stigmatization than are individual-oriented countries (e.g., Northern European/North American cultures).

## Conclusion

Stigma can be a barrier to testing behavior, to discovering infections, or to seeking treatment [9], such that increasing our understanding of stigma in the context of contagious infectious diseases, such as COVID-19, is important. Therefore, this study investigated the effect of disease-related and individual attributes of potentially stigmatized individuals in stigma formation, and familiarity with COVID-19 in potential stigmatizers, based on a large-scale factorial survey and two theoretical approaches. In line with attribution theory, our results reveal that individuals with COVID-19 (versus the flu) are stigmatized more by way of negative cognitions, affects, and behavioral inclinations. Also, additional signals and signaling events, such as having control over the onset of the disease—which evokes impressions of responsible or reckless behavior, putting others at risk of infection—increased such negative reactions.

Our results also suggest that predictions of the familiarity hypothesis about stigma-reducing effects of familiarity with COVID-19 only partially hold. While more factual knowledge about COVID-19 reduced all forms of stigma, self-reported knowledge and vicarious experience exacerbated most negative reactions towards others. Such a double-edged role of familiarity has been previously observed [81, 82], and the discrepancy between self-reported knowledge and factual knowledge can be envisioned as yet another instantiation of the Dunning–Kruger Effect. Thus, future research should aim at better understanding the nature of familiarity effects [e.g., 40], also in relation to the interaction between signals and signaling events and a person’s familiarity to further our understanding of the person–situation interaction [43–45]. This study also has practical implications in stigma intervention. The finding on the stigma-reducing effect of factual knowledge may offer an effective tool for developing interventions to combat the stigmatization of individuals infected by COVID-19. Providing facts on the pandemic in different channels has positive effects beyond promoting more responsible preventive behavior [83, 84]. These facts could also include information about the stigmatization of infected or potentially infected individuals, since stigma can delay diagnosis or treatment-seeking and thus lead to detrimental health and societal outcomes.

## Supplementary Information

**Additional file 1:** **Table S1.**

**Additional file 2:** **Table S2.**

## Data Availability

The data that support the findings of this study are openly available [85].
