# Supplementary material for: Stigmatization in the context of the COVID-19 pandemic: a survey experiment using attribution theory and the familiarity hypothesis
Source: BMC Public Health. 2023 Mar 18;23:521. doi: 10.1186/s12889-023-15234-5 (PMC10024019; doi:10.1186/s12889-023-15234-5)
Supplement: Supplementary file 2 — Additional file 2: Table S2. [file 12889_2023_15234_MOESM2_ESM.docx]

**Table S2.** Multivariate negative binomial regression models^†^ on stigma in the context of COVID-19—examining interaction effects between diagnosis with all other vignette characteristics and all indicators of familiarity (*N_Participants_*=4,059).

| **Model** |  | **1** | **2** |  | **3** | **4** |  | **5** | **6** | **7** |
| --- | --- | --- | --- | --- | --- | --- | --- | --- | --- | --- |
| **Stigma type** |  | **Negative cognition** | |  | **Negative affects** | |  | **Discriminatory inclinations** | | |
| **Stigma facet** |  | **Blame** | **Deservingness** |  | **No sympathy** | **Anger** |  | **Avoidance** | **Insulting** | **Triage** |
| **Vignette characteristics** |  |  |  |  |  |  |  |  |  |  |
| Diagnosis: COVID-19 (Ref. Flu) | *IRR* | 1.240 | 1.038 |  | 1.383 | 1.685 |  | 1.964 | 1.007 | 1.049 |
|  | *p* | .300 | .936 |  | .236 | .048* |  | .025* | .989 | .898 |
|  | *q* | .286 | .594 |  | .235 | .063 |  | .038* | .620 | .588 |
| Precipitating event: Visited friend | *IRR* | 1.661 | 1.896 |  | 1.686 | 1.728 |  | 1.710 | 1.541 | 1.522 |
| (Ref. Blank) | *p* | .000*** | .000*** |  | .000*** | .000*** |  | .000*** | .000*** | .000*** |
|  | *q* | .001** | .001** |  | .001** | .001** |  | .001** | .001** | .001** |
| Quarantine instruction: Violated | *IRR* | 1.453 | 1.606 |  | 1.892 | 2.336 |  | 1.620 | 1.873 | 1.579 |
| (Ref. Followed) | *p* | .000*** | .000*** |  | .000*** | .000*** |  | .000*** | .000*** | .000*** |
|  | *q* | .001** | .001** |  | .001** | .001** |  | .001** | .001** | .001** |
| Belonging to risk group: Yes | *IRR* | 1.017 | .952 |  | .984 | .977 |  | 1.048 | .980 | .955 |
| (Ref. None) | *p* | .712 | .616 |  | .786 | .682 |  | .461 | .845 | .564 |
|  | *q* | .513 | .478 |  | .533 | .513 |  | .404 | .570 | .445 |
| Risk area: Yes (Ref. No) | *IRR* | 1.060 | 1.136 |  | 1.061 | 1.023 |  | 1.136 | 1.117 | 1.011 |
|  | *p* | .197 | .202 |  | .310 | .690 |  | .047* | .289 | .892 |
|  | *q* | .204 | .204 |  | .294 | .513 |  | .063 | .279 | .588 |
| Gender: Female (Ref. Male) | *IRR* | .850 | .763 |  | .841 | .906 |  | .810 | .775 | .847 |
|  | *p* | .000*** | .007** |  | .003** | .080 |  | .001** | .014* | .039* |
|  | *q* | .001** | .013* |  | .007** | .099 |  | .003** | .024* | .054 |
| Age: Old (Ref. Young) | *IRR* | .995 | 1.041 |  | 1.006 | .976 |  | 1.081 | .918 | 1.065 |
|  | *p* | .904 | .681 |  | .922 | .657 |  | .226 | .404 | .434 |
|  | *q* | .588 | .513 |  | .591 | .501 |  | .226 | .359 | .379 |
| Origin: Foreigner (Ref. Local) | *IRR* | 1.040 | 1.051 |  | .918 | .962 |  | .954 | .969 | .974 |
|  | *p* | .381 | .616 |  | .143 | .488 |  | .464 | .759 | .737 |
|  | *q* | .356 | .478 |  | .159 | .411 |  | .405 | .515 | .515 |
| **Familiarity indicators** |  |  |  |  |  |  |  |  |  |  |
| Self-reported COVID-19 | *IRR* | 1.330 | 1.895 |  | 1.654 | 2.009 |  | 1.508 | 1.281 | 1.497 |
| knowledge | *p* | .026* | .027* |  | .002** | .000*** |  | .025* | .399 | .085 |
|  | *q* | .039* | .039* |  | .006** | .001** |  | .038* | .357 | .102 |
| COVID-19 knowledge test | *IRR* | .516 | .136 |  | .645 | .593 |  | .464 | .136 | .288 |
|  | *p* | .000*** | .000*** |  | .017* | .003** |  | .000*** | .000*** | .000*** |
|  | *q* | .001** | .001** |  | .027* | .006** |  | .001** | .001** | .001** |
| Vicarious experience (Ref. None) | *IRR* | 1.135 | .993 |  | 1.225 | 1.292 |  | 1.194 | 1.329 | 1.361 |
|  | *p* | .110 | .967 |  | .046* | .008** |  | .115 | .111 | .027* |
|  | *q* | .126 | .614 |  | .062 | .015* |  | .130 | .126 | .039* |
| **Further respondent characteristics** |  |  |  |  |  |  |  |  |  |  |
| Female (Ref. Male) | *IRR* | .885 | .718 |  | .875 | .974 |  | .924 | .748 | .740 |
|  | *p* | .000*** | .000*** |  | .001** | .501 |  | .079 | .000*** | .000*** |
|  | *q* | .001** | .001** |  | .003** | .413 |  | .099 | .001** | .001** |
| Age | *IRR* | .993 | .991 |  | .994 | .991 |  | .994 | .999 | .993 |
|  | *p* | .000*** | .000*** |  | .000*** | .000*** |  | .000*** | .742 | .000*** |
|  | *q* | .001** | .001** |  | .001** | .001** |  | .001** | .515 | .001** |
| **Diagnosis*Vignette characteristics** |  |  |  |  |  |  |  |  |  |  |
| Diagnosis*Precipitating event | *IRR* | 1.128 | .947 |  | 1.006 | .989 |  | .793 | 1.000 | 1.061 |
|  | *p* | .046* | .693 |  | .942 | .889 |  | .010** | .999 | .596 |
|  | *q* | .062 | .513 |  | .594 | .588 |  | .017* | .620 | .466 |
| Diagnosis*Quarantine instruction | *IRR* | .778 | 1.040 |  | .886 | .799 |  | .820 | .916 | .992 |
|  | *p* | .000*** | .776 |  | .138 | .004** |  | .027* | .546 | .943 |
|  | *q* | .001** | .526 |  | .154 | .008** |  | .039* | .441 | .594 |
| Diagnosis*Belonging to risk | *IRR* | 1.023 | 1.124 |  | 1.090 | 1.102 |  | 1.120 | 1.133 | 1.080 |
| group | *p* | .709 | .396 |  | .290 | .204 |  | .205 | .389 | .491 |
|  | *q* | .513 | .357 |  | .279 | .205 |  | .205 | .356 | .411 |
| Diagnosis*Risk area | *IRR* | .944 | .929 |  | 1.021 | 1.061 |  | .895 | .903 | .999 |
|  | *p* | .342 | .595 |  | .797 | .437 |  | .213 | .478 | .989 |
|  | *q* | .326 | .466 |  | .541 | .380 |  | .213 | .409 | .620 |
| Diagnosis*Gender | *IRR* | 1.212 | 1.249 |  | 1.177 | 1.045 |  | 1.153 | 1.089 | 1.110 |
|  | *p* | .001** | .109 |  | .046* | .564 |  | .112 | .556 | .350 |
|  | *q* | .004** | .126 |  | .062 | .445 |  | .126 | .442 | .334 |
| Diagnosis*Age | *IRR* | 1.015 | .885 |  | 1.100 | 1.036 |  | .972 | 1.056 | .960 |
|  | *p* | .805 | .376 |  | .241 | .647 |  | .754 | .705 | .715 |
|  | *q* | .541 | .356 |  | .238 | .498 |  | .515 | .513 | .513 |
| Diagnosis*Origin | *IRR* | .908 | .980 |  | 1.059 | .971 |  | 1.031 | .864 | .983 |
|  | *p* | .110 | .882 |  | .480 | .701 |  | .734 | .310 | .875 |
|  | *q* | .126 | .588 |  | .409 | .513 |  | .515 | .294 | .588 |
| **Diagnosis*Familiarity indicators** |  |  |  |  |  |  |  |  |  |  |
| Diagnosis*Self-reported | *IRR* | 1.016 | .565 |  | .682 | .774 |  | .843 | 1.062 | .912 |
| COVID-19 knowledge | *p* | .927 | .142 |  | .090 | .236 |  | .489 | .881 | .773 |
|  | *q* | .591 | .159 |  | .109 | .235 |  | .411 | .588 | .526 |
| Diagnosis*COVID-19 knowledge | *IRR* | 1.684 | 1.747 |  | 1.032 | 1.104 |  | .827 | 1.181 | 1.189 |
| test | *p* | .007** | .193 |  | .905 | .692 |  | .510 | .708 | .624 |
|  | *q* | .014* | .203 |  | .588 | .513 |  | .421 | .513 | .483 |
| Diagnosis*Vicarious experience | *IRR* | .868 | 1.094 |  | .929 | .844 |  | .900 | 1.081 | .845 |
|  | *p* | .179 | .708 |  | .600 | .199 |  | .499 | .751 | .383 |
|  | *q* | .190 | .513 |  | .466 | .204 |  | .413 | .515 | .356 |
| **Anonymity perceptions** | *IRR* | 1.003 | .913 |  | .961 | 1.003 |  | .976 | .882 | .945 |
|  | *p* | .801 | .001*** |  | .009** | .839 |  | .163 | .000*** | .009** |
|  | *q* | .541 | .003** |  | .017* | .567 |  | .177 | .001** | .017* |
| **Constant** | *IRR* | 2.893 | 6.507 |  | 2.166 | 1.346 |  | 2.318 | 6.872 | 5.349 |
|  | *p* | .000*** | .000*** |  | .000*** | .145 |  | .000*** | .000*** | .000*** |
|  | *q* | .001** | .001** |  | .001** | .159 |  | .001** | .001** | .001** |

***Notes:*** ^†^Incidence rate ratios (sharpened false discovery rate-adjusted *q*-value in parentheses). **p & q*<.05; **<0.01; ***<0.001 (two-tailed).
